# Shear wave elastography assessment of periventricular white matter and thalamic stiffness changes in infants with congenital heart disease undergoing cardiac surgery

**DOI:** 10.3389/fmed.2026.1841401

**Published:** 2026-09-11

**Authors:** Liping Sun, Wei Ji, Kan Zhang, Hualin Chen, Hong Wang, Jijian Zheng, Yue Huang

**Affiliations:** 1 Department of Anesthesiology, Shanghai Children’s Medical Center, Shanghai Jiao Tong University School of Medicine, Shanghai, China; 2 Department of Anesthesiology, National Children’s Medical Center, Shanghai, China; 3 GE Healthcare, Shanghai, China

**Keywords:** congenital heart disease, infants, periventricular white matter, shear wave elastography, thalamus

## Abstract

**Objective:**

To quantitatively evaluate stiffness changes in the periventricular white matter (PVWM) and thalamus (TH) in infants undergoing congenital cardiac surgery using shear wave elastography (SWE).

**Design:**

A prospective observational study.

**Setting:**

Single-center study based at a tertiary care center.

**Patients:**

Forty-three infants aged zero-to-six months with congenital heart disease (CHD) undergoing cardiac surgery with cardiopulmonary bypass (CPB) were enrolled.

**Interventions:**

SWE was performed to measure periventricular white matter and thalamic stiffness before and after surgery.

**Measurements and Main Results:**

No significant correlation was observed between age and the stiffness of either PVMW or TH, both before and after surgery. Significant postoperative reductions were observed in mean stiffness of PVMW (1.22 ± 0.14 m/s vs. 1.18 ± 0.14 m/s, *p* = 0.014) and TH (1.54 ± 0.15 m/s vs. 1.50 ± 0.15 m/s, *p* = 0.031). Regional analysis of PVWM and TH indicated a significant postoperative decrease in tissue stiffness, with notable reductions observed in the body portion of PVWM (1.24 ± 0.13 m/s vs. 1.18 ± 0.15 m/s, *p* = 0.046), the caudal portion of PVWM (1.22 ± 0.14 m/s vs. 1.18 ± 0.13 m/s, *p* = 0.045), and the caudal portion of TH (1.53 ± 0.16 m/s vs. 1.45 ± 0.16 m/s, *p* = 0.014). Consistently, PVWM demonstrated lower stiffness than TH, both before surgery (1.22 ± 0.14 m/s vs. 1.54 ± 0.15 m/s, *p* < 0.001) and after surgery (1.18 ± 0.14 m/s vs. 1.50 ± 0.15 m/s, *p* < 0.001).

**Conclusions:**

SWE identified significant postoperative reductions in the stiffness of PVWM and TH in infants with CHD. The body and caudal PVWM, as well as caudal TH, showed the most prominent changes. PVWM consistently presented lower stiffness than TH during the entire perioperative period. SWE proves feasible for quantitative evaluation of minor cerebral changes in affected infants during the perioperative period.

## Introduction

Perioperative management and surgical techniques for congenital heart disease (CHD) have advanced substantially, leading to remarkable improvements in long-term survival among affected children. More than 90% of patients with simple CHD currently reach adulthood (1, 2). Despite improved cardiac prognosis, neurodevelopmental impairment remains a common and persistent adverse outcome associated with CHD. Over half of adolescents and young adults with moderate or complex CHD suffer from long-term neurocognitive and psychiatric sequelae, seizures, and audiovisual deficits, which substantially impair their long-term quality of life (3–7).

Preoperative and postoperative brain white matter injury (WMI) constitutes the primary pathological basis for neurodevelopmental deficits in infants with CHD. Cranial magnetic resonance imaging (MRI) studies have consistently demonstrated a high prevalence of perioperative brain injury in this vulnerable pediatric population. Prospective data indicate that preoperative WMI occurs in approximately 20% of CHD infants, whereas cardiopulmonary bypass (CPB) assisted cardiac surgery further increases the rate of new postoperative WMI to 44% (8). Accumulating evidence suggests that preexisting silent brain lesions do not progress after cardiac surgery, indicating that newly acquired perioperative injury is the predominant contributor to adverse long-term neurodevelopmental outcomes (9). Therefore, early detection and continuous real-time monitoring of perioperative cerebral damage are essential for optimizing perioperative strategies and reducing the overall burden of brain injury in CHD infants (8, 9).

Although MRI is regarded as the gold standard for diagnosing neonatal brain injury, its utility for routine perioperative bedside monitoring is considerably limited. MRI requires dedicated magnetic shielding environments and prolonged patient transportation, which is logistically challenging and potentially hazardous for critically ill postoperative infants. This unmet clinical need necessitates a rapid, safe, bedside-accessible, and quantitative tool for real-time intraoperative cerebral monitoring during congenital cardiac surgery.

Cranial ultrasound serves as the first-line bedside imaging modality for neonatal brain screening due to its noninvasiveness, real-time imaging capability, and favorable intraoperative compatibility. It allows continuous perioperative evaluation of cerebral hemodynamics and enables individualized regulation of cerebral perfusion. However, conventional two-dimensional ultrasound only provides qualitative or semiquantitative lesion assessment with high operator dependency, while Doppler ultrasound merely quantifies cerebral blood flow without characterizing the severity of parenchymal pathological changes. These drawbacks limit their ability to identify subtle perioperative cerebral alterations precisely and dynamically.

Shear wave elastography (SWE) is an advanced ultrasound technique that generates transverse shear waves via focused acoustic radiation force. The propagation velocity of shear wave is converted into color-coded elastograms and quantitative Young's modulus values, which directly reflect tissue stiffness (10). Although widely applied in the evaluation of hepatic, thyroid and breast lesions (11–13), SWE remains underutilized in neuroimaging research. In 2018, two-dimensional SWE was first applied to assess brain parenchymal stiffness in term and preterm neonates. This work validated the feasibility of transfontanellar SWE for neonatal brain assessment and established reference stiffness values of 8.28 kPa for the thalamus (TH) and 6.59 kPa for the periventricular white matter (PVWM) in healthy infants (14). Subsequent research confirmed a positive correlation between brain tissue stiffness and intracranial pressure (ICP) in pediatric patients with hydrocephalus (15). A recent study by Faure et al. confirmed excellent agreement between transfontanellar SWE and MRI for quantitative assessment of neonatal white matter damage. Damaged white matter showed higher stiffness than normal tissue, validating the clinical utility of SWE for evaluating neonatal brain injury (16).

Cerebral stiffness represents a complex biomechanical property modulated by multiple microstructural and physiological factors, including cellular density, myelination degree, cerebral edema, blood perfusion and ICP (15, 17–20). Perioperative brain injury is a common complication in infants with CHD, predominantly involving TH and PVWM. Such injuries are mainly attributed to CHD-related chronic hypoxia, CPB-triggered inflammation, surgical trauma, and impaired cerebral autoregulation induced by anesthetic and vasoactive medications (21–24). Notably, existing pediatric and neonatal studies have reported conflicting findings regarding stiffness alterations following acute brain injury: ischemic edema and demyelination generally reduce tissue stiffness, while intracranial hemorrhage, elevated ICP and reactive gliosis tend to increase stiffness (25–28). To date, no consensus has been reached on the correlation between stiffness changes and acute cerebral damage. Furthermore, SWE measurements are vulnerable to multiple confounding factors, such as sedation depth, fontanelle tension and probe compression, which may cause fluctuations in stiffness values irrelevant to organic brain lesions (29–31).

Given the existing controversies and insufficient evidence regarding perioperative cerebral stiffness changes, the present exploratory study was designed. We utilized SWE to quantitatively characterize perioperative stiffness changes of PVWM and TH in infants undergoing congenital cardiac surgery, aiming to explore the feasibility of SWE for detecting and monitoring cerebral parenchymal stiffness variation throughout the perioperative period.

## Materials and methods

### Ethics

This study was approved by the Ethics Committee of Shanghai Children's Medical Center on May 22, 2025 (Study title: Application of Shear Wave Elastography for Brain Parenchyma Assessment in Pediatric Patients with Congenital Heart Disease During the Perioperative Period, Ethical Approval No. SCMCIRB-K2025117-1). It was subsequently registered in the Chinese Clinical Trial Registry (Registration No.: ChiCTR2500103442) on May 29, 2025. All procedures performed in this study were in accordance with the 1975 Declaration of Helsinki and the relevant ethical regulations of Shanghai Children's Medical Center.

## Patients

Screening was conducted from May 2025 to August 2025 for infants aged 0 to 6 months with CHD undergoing elective cardiac surgery. All study subjects were confirmed as full-term infants on preoperative evaluation, and preterm infants were excluded to standardize baseline brain developmental status. Before surgery, written informed consent was obtained from the legal guardians of the eligible children. Patients were excluded if they met any of the following criteria: those requiring emergency surgery, those with an American Society of Anesthesiologists Physical Status Classification (ASA) > III, and those with a poor ultrasonic signal from the anterior fontanelle.

## Anesthesia protocol

All enrolled pediatric patients received oral midazolam at a dose of 0.5 mg/kg 20–30 min before anesthesia induction. For preoxygenation, 100% oxygen was inhaled prior to induction. Subsequently, intravenous anesthesia induction was performed with midazolam (0.05–0.1 mg/kg), etomidate (0.2–0.3 mg/kg), sufentanil (1.0–2.0 µg/kg), and rocuronium (0.6–1.0 mg/kg). After induction, tracheal intubation was implemented via video laryngoscopy using a cuffed endotracheal tube with appropriate size. All children received pressure-controlled mechanical ventilation with an inspired oxygen concentration of 50%. The tidal volume was set at 8–10 mL/kg, and the ventilation frequency and inspiratory pressure were dynamically adjusted to maintain the end-tidal carbon dioxide partial pressure (ETCO_2_) at 32–38 mmHg during the scanning period.

Intravenous anesthesia was continuously maintained with propofol (4.0–6.0 mg/kg/h), sufentanil (2.0–2.5 µg/kg/h), and rocuronium (0.5 mg/kg/h) throughout the post-induction scanning and perioperative period.

## Two-dimensional SWE measurements

All enrolled infants underwent two cranial ultrasonography assessments: the first scan was within 5 min after anesthetic induction in the operating room, and the second scan was obtained 24 h postoperatively in the cardiac intensive care unit. All infants were examined in the supine position via the anterior fontanelle. Conventional grayscale B-mode imaging was initially performed to visualize cerebral anatomical structures and define standardized sagittal scanning planes, followed by SWE acquisition. Both grayscale ultrasound and SWE measurements were acquired using a Logic-e Next Gen ultrasound system (GE Healthcare, Wauwatosa, WI) equipped with a C1-6-D XDclear 1-6 MHz convex transducer. All ultrasound examinations were performed by a single experienced anesthesiologist proficient in pediatric cranial ultrasound, who had completed more than 100 relevant infant examinations and was fully blinded to patients' surgical information and clinical outcomes.

To evaluate intra-observer reproducibility, 20% of randomly selected scans were re-analyzed by the same operator two weeks after the initial evaluation. The intra-class correlation coefficients (ICCs) for all measured brain regions were 0.88 (95% CI: 0.82-0.93), indicating excellent reproducibility and reliability of SWE measurements. All quantitative analyses were independently performed by another researcher who was fully blinded to group allocation and clinical data.

**Figure 1:**
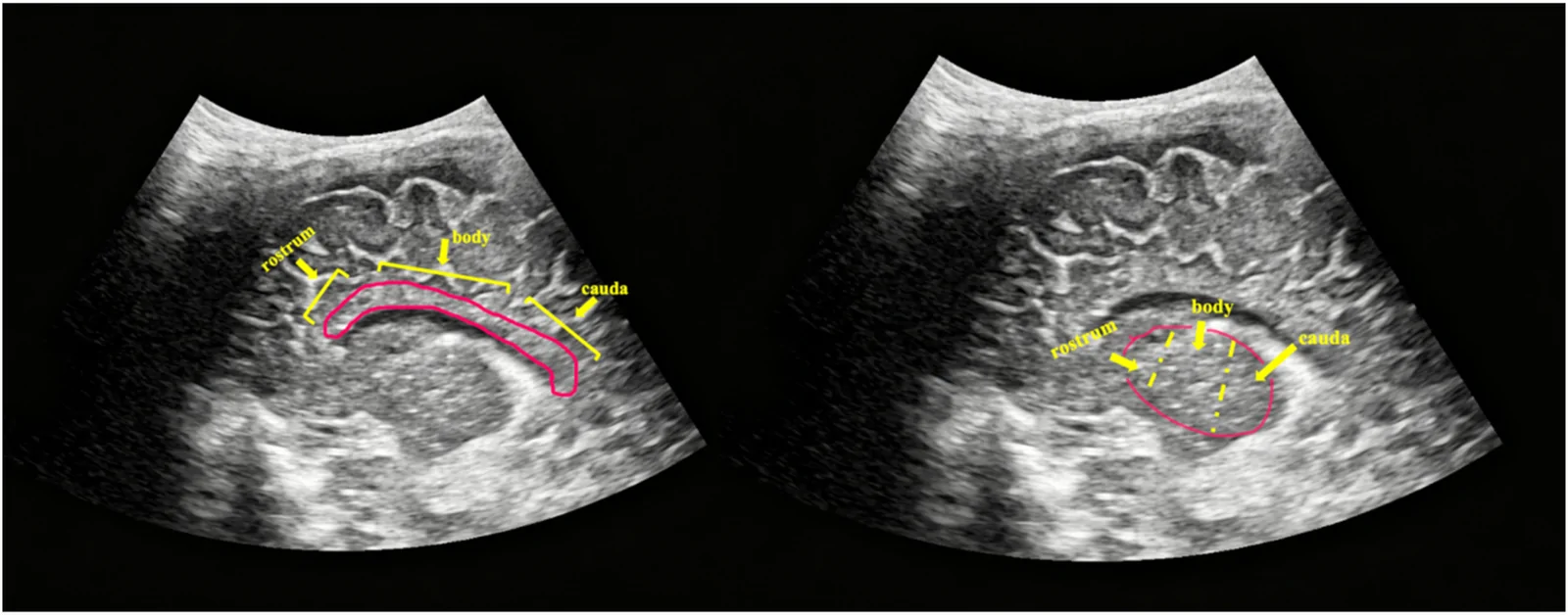
Anatomical segmentation of PVWM and thalamus into rostrum, body, and cauda in the sagittal view.

SWE measurements were targeted at PVWM and TH. All SWE evaluations were acquired in the sagittal plane. Both PVWM and TH were further divided into three segments for separate analysis: rostrum, body, and cauda. For PVWM, the rostrum referred to the anterior PVWM adjacent to the frontal horn of the lateral ventricle; the body corresponded to PVWM alongside the body of the lateral ventricle; and the cauda represented PVWM near the atrium and occipital horn. For TH, the rostrum was defined as the anterior thalamic nuclear complex, the body as the central and lateral nuclear groups, and the cauda as the pulvinar region. Figure 1 present a labeled schematic diagram illustrating the segmentation of PVWM and TH with unified anatomical markers for SWE measurements regions.

**Figure 2:**
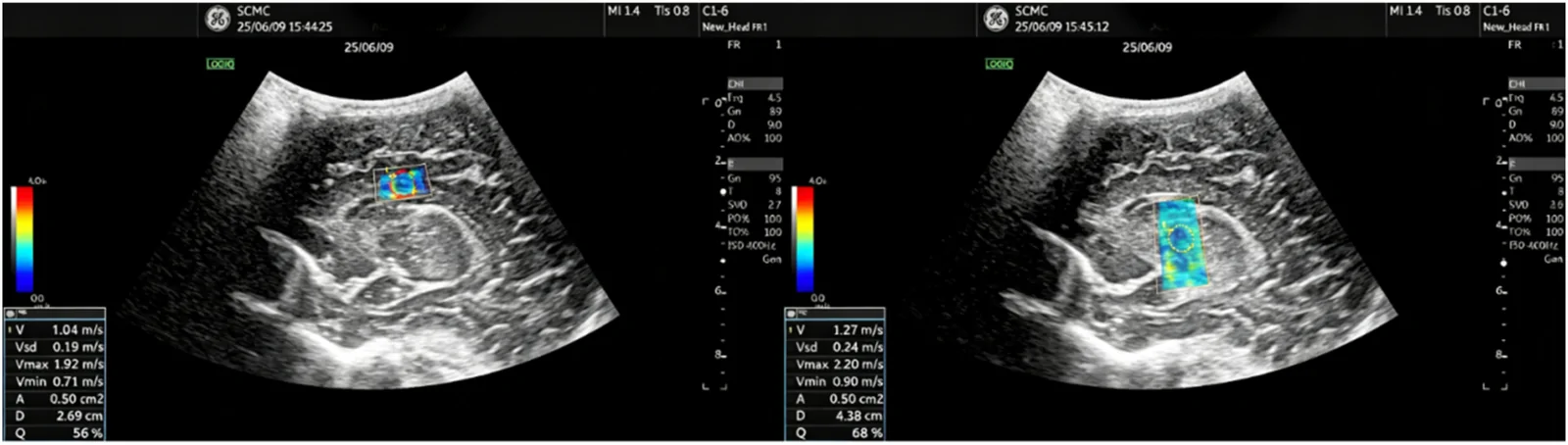
B-mode cranial ultrasound images with shear wave elastography measurements.

To guarantee accurate and objective quantification of elastic parameters, the operator manipulated the equipment gently apply and placed the probe lightly on the cranial surface. Particular care was taken to avoid vascular structures during the measurement process. A rectangular box was placed on the measurement area, and the SWE images were obtained in the form of homogeneous coloring that filled the rectangle (Figure 2). Subsequently, circular regions of interest (ROIs) approximately 0.5 cm^2^ in diameter were placed within the rectangular area to quantify tissue stiffness. The system automatically excluded ROIs that extended beyond the elastogram boundaries or contained unfilled pixels (invalid measurements), and the mean shear wave velocity within the valid ROIs was recorded in meters per second (m/s) (both m/s and kPa are conventional units for SWE assessments in clinical research). For each patient and each brain region, three separate acquisitions with quantitative measurements were performed. The average value was calculated and used for statistical analysis.

This is the first study to apply SWE for perioperative cerebral assessment in infants with CHD. All SWE measurements were performed on the left side only. This standardized unilateral scanning protocol was implemented to reduce operational errors, maintain data consistency, and shorten scanning time in anesthetized infants.

## Statistical analysis

**Figure 3:**
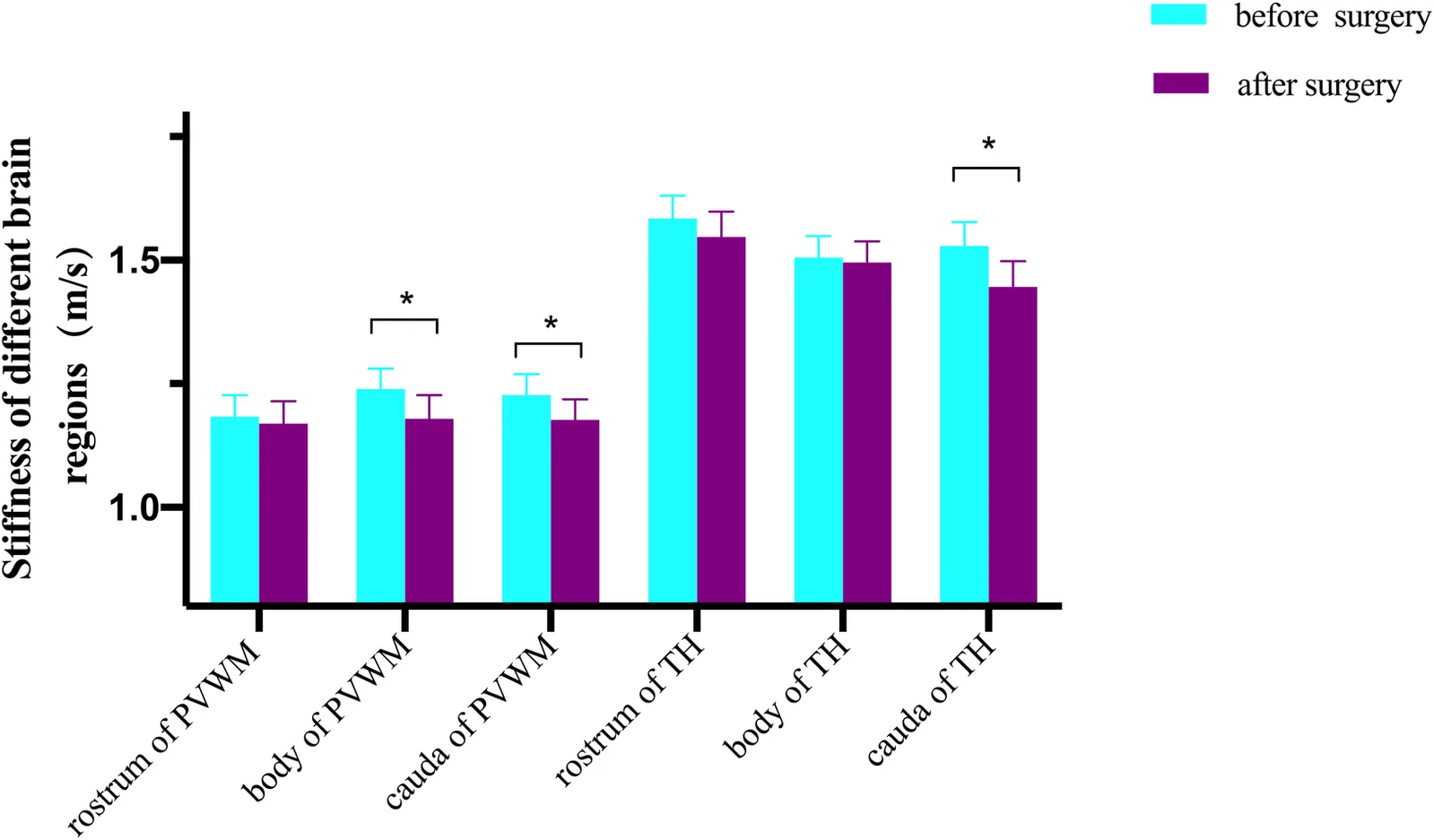
Different stiffness of brain regions. PVWM, periventricular white matter; TH, thalamus.

Statistical analyses were performed with IBM SPSS Statistics 23 (IBM Corporation, Armonk, NY). Continuous data are presented as mean ± standard deviation. This study adopted a paired study design. Paired-samples t-tests were applied to compare perioperative stiffness of PVWM and TH, including the whole regions and their three subregions (rostrum, body and cauda) (Table 1). The same test was also used to compare stiffness between PVWM and TH at two time points (Figure 3). Each brain region was measured three times via SWE. The average of the three replicates was calculated to reduce measurement bias and used for final statistical analyses, and all continuous variables followed a normal distribution.

**Table 1:** Stiffness differences in periventricular white matter and thalamus before and after surgical intervention. PVWM, periventricular white matter; TH, thalamus.

Region		Before surgery	After surgery	*p*
PVWM (m/s)	all	1.22 ± 0.14	1.18 ± 0.14	0.014
	rostrum	1.18 ± 0.14	1.17 ± 0.14	0.755
	body	1.24 ± 0.13	1.18 ± 0.15	0.046
	cauda	1.22 ± 0.14	1.18 ± 0.13	0.045
TH (m/s)	all	1.54 ± 0.15	1.50 ± 0.15	0.031
	rostrum	1.58 ± 0.15	1.55 ± 0.16	0.279
	body	1.51 ± 0.14	1.50 ± 0.13	0.938
	cauda	1.53 ± 0.16	1.45 ± 0.16	0.014

All preoperative vs. postoperative comparisons were analyzed using paired-samples t-tests. Three repeated measurements per region were averaged for statistical analysis. Marginal *P*-values of 0.045 and 0.046 would lose statistical significance after multiple comparison correction. PVWM, periventricular white matter; TH, thalamus.

Given the exploratory nature of this pilot study, all results are reported using unadjusted *P* values. Multiple comparisons could increase the risk of Type I error. Particularly, the marginal *P* values of 0.045 and 0.046 in Table 1 would lose statistical significance after multiple comparison correction, which warrants cautious interpretation. Pearson correlation analysis was used to explore correlations between infant age and PVWM/TH stiffness at preoperative and postoperative time points respectively. Statistical significance was set at two-tailed *P* < 0.05.

## Results

This pilot study initially screened 85 infants with CHD, among whom 45 met the inclusion criteria and were enrolled. Two infants discontinued treatment due to inadequate acoustic windows, resulting in 43 completed studies and statistical analysis. The demographic, clinical and above surgical characteristics of the study participants are presented in Table 2.

**Table 2:** Baseline characteristics of the included patients.

All patients (*n* = 43)	
Age (months)	3.3 ± 1.9
Sex (male: female)	22:21
Weight (kg)	5.6 ± 1.5
Height (cm)	59.6 ± 5.9
Duration of CPB, mins	57.5 (37–83.8)
Aortic cross-clamp time, mins	31.0 (21.0–41.0)
Mild hypothermia, %	34.9
Moderate hypothermia, %	9.3
Deep hypothermia, %	2.3
Diagnosis	ASD (*n* = 6), VSD (*n* = 8), VSD + ASD (*n* = 11), IAA (*n* = 1), TAPVC (*n* = 4), TOF (*n* = 6), TGA (*n* = 1), DORV (*n* = 1), PA (*n* = 1), COA (*n* = 3), PDA (*n* = 1)

Non-normothermic cooling includes mild hypothermia, moderate hypothermia and deep hypothermia. No additional cooling means no intraoperative hypothermia management was performed. CPB, cardiopulmonary bypass; ASD, atrial septal defect; VSD, ventricular septal defect; IAA, interrupted aortic arch; TAPVC, total anomalous pulmonary venous connection; TOF, tetralogy of fallot; TGA, transposition of the great arteries; DORV, double outlet right ventricle; PA, pulmonary atresia; COA, coarctation of the aorta; PDA, patent ductus arteriosus.

Compared with preoperative measurements, the overall postoperative stiffness of PVWM significantly decreased (1.22 ± 0.14 m/s vs. 1.18 ± 0.14 m/s, *p* = 0.014). Subregional analysis showed significant stiffness reductions in both PVWM body (1.24 ± 0.13 m/s to 1.18 ± 0.15 m/s, *p* = 0.046) and PVWM caudal (1.22 ± 0.14 m/s to 1.18 ± 0.13 m/s, *p* = 0.045). Similarly, the overall TH stiffness was significantly lower postoperatively than preoperatively (1.54 ± 0.15 m/s vs. 1.50 ± 0.15 m/s, *p* = 0.031), with the most prominent decrease observed in TH caudal (1.53 ± 0.16 m/s to 1.45 ± 0.16 m/s, *p* = 0.014). (Table 1, Figure 3).

**Figure 4:**
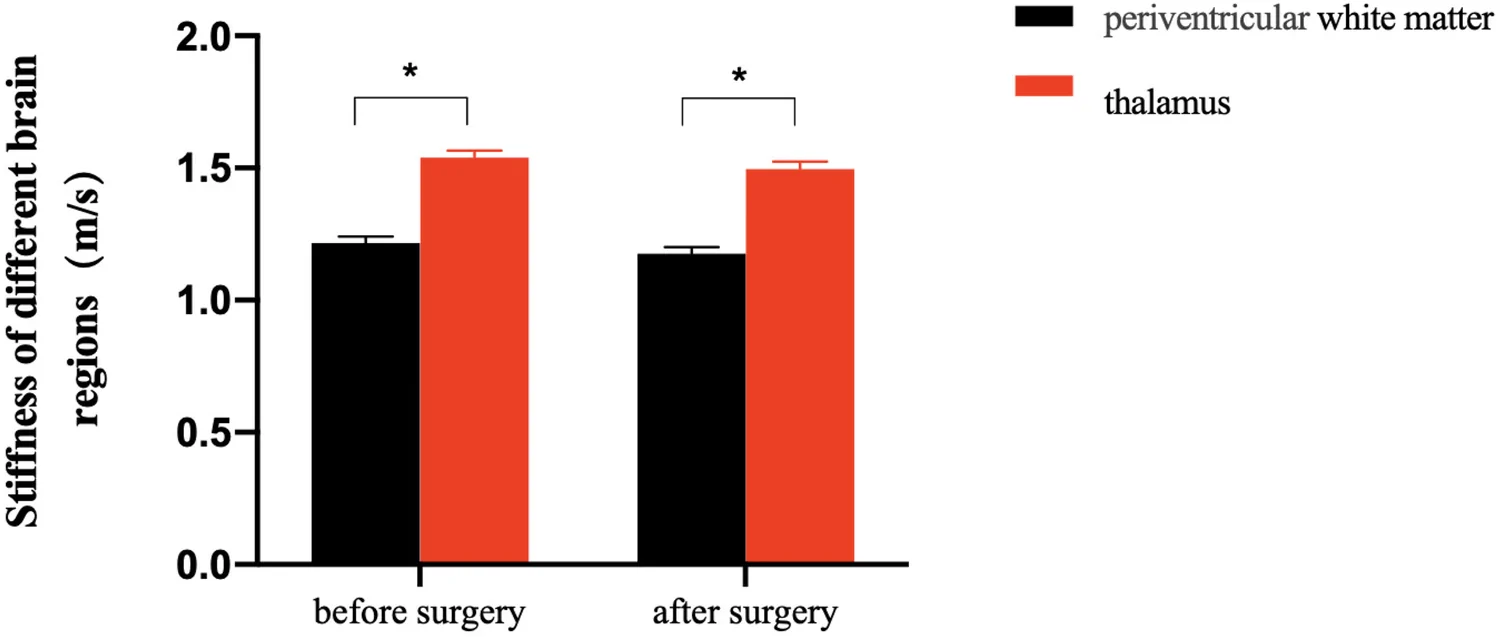
Comparison of stiffness between periventricular white matter and thalamus at preoperative and postoperative time points. Inter-group comparisons at each time point were performed using paired-samples t-tests.

At both preoperative and postoperative time points, PVWM exhibited significantly lower tissue stiffness than TH (preoperative: 1.22 ± 0.14 m/s vs. 1.54 ±  0.15 m/s, *p* < 0.001; postoperative: 1.18 ±  0.14 m/s vs. 1.50 ±  0.15 m/s, *p* < 0.001) (Figure 4).

**Figure 5:**
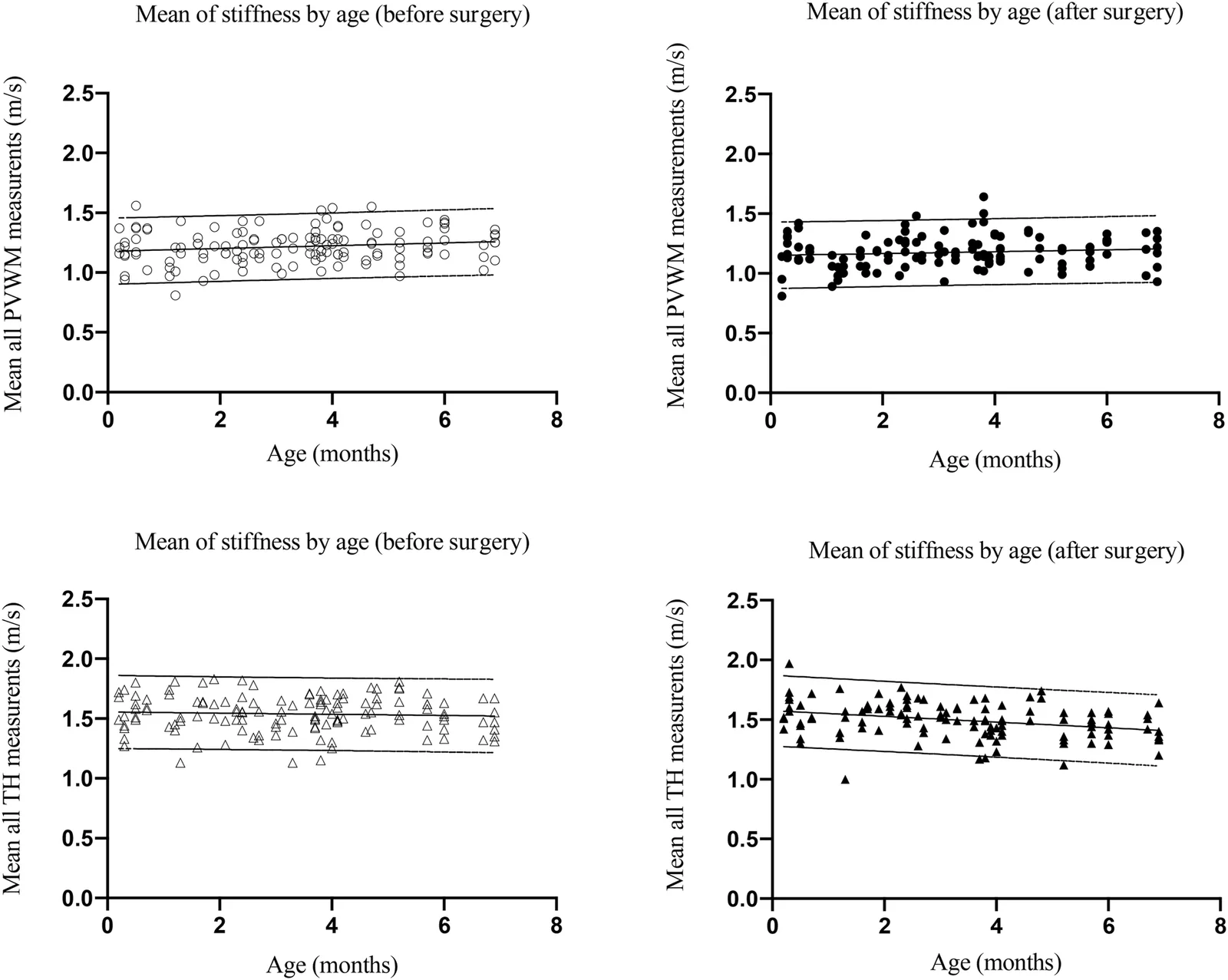
Distribution of the mean values of all measurements and the prediction interval by age. PVWM, periventricular white matter; TH, thalamus.

Correlation analysis revealed no significant linear association between infant age and PVWM or TH stiffness at baseline. Specifically, no significant correlations were found between age and preoperative PVWM stiffness (r = 0.031, *p* = 0.842) or preoperative TH stiffness (r = 0.02, *p* = 0.9). For postoperative measurements, age was not correlated with TH stiffness (r = −0.061, *p* = 0.716), while a marginal positive trend was observed between age and postoperative PVWM stiffness (r = 0.302, *p* = 0.065). This borderline trend likely resulted from the limited statistical power of this small-sample exploratory pilot study (*n* = 43) (Figure 5).

## Discussion

This study used SWE to evaluate perioperative changes in tissue stiffness of the PVWM and TH in infants with CHD. Our results demonstrate that in infants with CHD, the stiffness of PVWM is significantly lower than that of TH. Following cardiac surgery, both PVWM and TH exhibit a marked reduction in stiffness compared to preoperative levels. However, no significant correlation was observed between stiffness (in either PVWM or TH) and age among infants with CHD.

Our results are consistent with a previous report by McIlvain et al., who found no obvious age-related alterations in cerebral stiffness among children aged 5 to 7 years (32). Although a weak age-related trend was observed for postoperative PVWM stiffness, it did not reach statistical significance. All enrolled infants were full term infants. Accumulated evidence indicates that brain injury in infants with CHD can occur during the prenatal period and before surgery, and its severity varies across different CHD types. This may explain why brain stiffness was not associated with age in our cohort.

Postoperative brain injury is highly prevalent in children with CHD, while its specific mechanisms and time course remain unclear (21). PVWM is widely recognized as a major vulnerable site for brain injury in CHD patients (22, 23). In our cohort, PVWM stiffness decreased significantly from 1.22 ± 0.14 to 1.18 ± 0.14 after surgery (*p* < 0.05). Although oligodendrocyte precursor cells within PVWM are known to be highly vulnerable to hypoxia- and inflammation-mediated stress, the biological meaning of this postoperative stiffness shift cannot be definitively established in the present study (21). TH is also susceptible to neurological damage in children with CHD (24). Similarly, TH stiffness dropped significantly postoperatively from 1.54 ± 0.15 to 1.50 ± 0.15 (*p* < 0.05), suggesting that both white and gray matter sustain biomechanical damage after cardiac surgery.

We further divided PVWM and TH into rostrum, body, and cauda segments for subregional analysis. The most prominent postoperative stiffness reduction was seen in the body and caudal portions of PVWM, as well as caudal TH. These anatomical areas are adjacent to the choroid plexus, where inflammatory mediators can easily diffuse into surrounding neural tissues and later tissue structure (33).

Moreover, within our cohort of infants with CHD, PVWM showed lower stiffness compared with TH in the same subjects, which is consistent with previous report (14). This difference can be attributed to the distinct histological composition of these regions: TH exhibits characteristically higher stiffness due to its dense aggregation of neuronal cell bodies and neuropil. In comparison, white matter displays comparatively lower stiffness resulting from its myelin-rich lipid content and more compliant axonal architecture (24). These observations are further supported by established neurodevelopmental principles, which describe a centro-peripheral maturation pattern wherein TH, as a phylogenetically older structure, achieves structural maturity earlier than adjacent white matter tracts (34, 35).

Several limitations of this exploratory study need to be addressed. First, the between-group differences in shear wave velocity were small (0.04–0.08 m/s) and overlapped with standard deviations (0.13–0.16 m/s). Given that SWE is newly applied in this clinical setting, these subtle differences cannot be used as reliable diagnostic indicators. Second, no overt neurological complications were detected during hospitalization. Nevertheless, most perioperative brain injury in infants with CHD is subclinical, so silent brain damage cannot be ruled out. Third, not all patients underwent synchronous cranial MRI. Considering the risk of patient transfer and sedation, routine postoperative MRI is not performed in our department, which prevents correlation analysis between SWE measurements and MRI findings. Fourth, only left-sided measurements were acquired. This unilateral protocol was designed to simplify operation and shorten scanning time for this preliminary study, and bilateral detection will be adopted in further research. Fifth, no subgroup analyses were conducted for healthy controls or CHD subtypes. Additionally, the relatively small sample size precluded multivariate adjustment to avoid statistical overfitting. Sixth, the sedation state differed significantly between the two scanning time points. Baseline SWE measurements were obtained under full surgical general anesthesia, whereas the 24-hour postoperative scans were performed under routine ICU sedation and analgesia. Preclinical studies have confirmed that cerebral stiffness is highly sensitive to anesthesia depth and arousal level (36). The inconsistent sedation status at the two time points may interfere with the longitudinal comparison of brain tissue stiffness before and after surgery and constitutes an unavoidable confounding factor in this preliminary study. Large-sample prospective studies are therefore warranted to clarify the clinical implications of minor cerebral stiffness changes, combine MRI data for multimodal analysis, conduct bilateral assessments, and explore the influence of different CHD phenotypes. Long-term neurodevelopmental follow-up is also required to define the value of SWE in monitoring postoperative cerebral changes.

In conclusion, SWE can quantitatively assess the stiffness of PVWM and TH in infants with CHD during the perioperative period. The stiffness of these brain regions decreased after surgery, with the most pronounced changes observed in body and caudal parts of PVWM as well as caudal TH. PVWM consistently showed lower stiffness than TH throughout the entire perioperative phase. SWE enables quantitative monitoring of perioperative cerebral biomechanical properties and shows promising feasibility in this high-risk infant population; nevertheless, the clinical interpretation of subtle stiffness alterations requires further validation.

## Data Availability

The original contributions presented in the study are included in the article/Supplementary Material, further inquiries can be directed to the corresponding authors.

## References

[1] Moore JP Marelli A Burchill LJ Chubb H Roche SL Cedars AM. Management of heart failure with arrhythmia in adults with congenital heart disease. JACC state-of-the-art review. J Am Coll Cardiol. (2022) 80(23):2224–38. 10.1016/j.jacc.2022.09.038 36456053

[2] Buber J Valente AM. Predicting survival in adults with congenital heart disease: what are the odds? Heart. (2018) 104(20):1643–4. 10.1136/heartjnl-2018-312975 29666177

[3] Cabrera-Mino C DeVon HA Aboulhosn J Brecht ML Choi KR Pike NA. Neurocognition in adults with congenital heart disease post-cardiac surgery: a systematic review. Heart Lung. (2024) 64:62–73. 10.1016/j.hrtlng.2023.11.011 38043432

[4] Marelli A Miller SP Marino BS Jefferson AL Newburger JW. Brain in congenital heart disease across the lifespan: the cumulative burden of injury. Circulation. (2016) 133(20):1951–62. 10.1161/CIRCULATIONAHA.115.019881 27185022 PMC5519142

[5] Petit CJ Rome JJ Wernovsky G Mason SE Shera DM Nicolson SC. Preoperative brain injury in transposition of the great arteries is associated with oxygenation and time to surgery, not balloon atrial septostomy. Circulation. (2009) 119(5):709–16. 10.1161/CIRCULATIONAHA.107.760819 19171858 PMC2736632

[6] Donofrio MT Massaro AN. Impact of congenital heart disease on brain development and neurodevelopmental outcome. Int J Pediatr. (2010) 2010:359390. 10.1155/2010/359390 20862365 PMC2938447

[7] Wernovsky G Licht DJ. Neurodevelopmental outcomes in children with congenital heart disease- what can we impact? Pediatr Crit Care Med. (2016) 17(8 Suppl 1):S232–42. 10.1097/PCC.0000000000000800 27490605 PMC4975480

[8] Beca J Gunn JK Coleman L Hope A Reed PW Hunt RW. New white matter brain injury after infant heart surgery is associated with diagnostic group and the use of circulatory arrest. Circulation. (2013) 127:971–9. 10.1161/CIRCULATIONAHA.112.001089 23371931

[9] Block AJ McQuillen PS Chau V Glass H Poskitt KJ Barkovich AJ. Clinically silent preoperative brain injuries do not worsen with surgery in neonates with congenital heart disease. J Thorac Cardiovasc Surg. (2010) 140:5507. 10.1016/j.jtcvs.2010.03.035 PMC2917479 20434174

[10] Gennisson JL Deffieux T Fink M Tanter M. Ultrasound elastography: principles and techniques. Diagn Interv Imaging. (2013) 94:487–95. 10.1016/j.diii.2013.01.022 23619292

[11] Sandrin L Fourquet B Hasquenoph J-M Yon S Fournier C Mal F. Transient elastography: a new noninvasive method for assessment of hepatic fibrosis. Ultrasound Med Biol. (2003) 29:1705–13. 10.1016/j.ultrasmedbio.2003.07.001 14698338

[12] Vorlander C Wolff J Saalabian S Lienenluke RH Wahl RA. Real-time ultrasound elastography—a noninvasive diagnostic procedure for evaluating dominant thyroid nodules. Langenbecks Arch Surg. (2010) 395:865–71. 10.1007/s00423-010-0685-3 20632029

[13] Sigrist RMS Liau J Kaffas AE Chammas MC Willmann JK. Ultrasound elastography: review of techniques and clinical applications. Theranostics. (2017) 7(5):1303–29. Published 2017 Mar 7 10.7150/thno.18650 28435467 PMC5399595

[14] Albayrak E Kasap T. Evaluation of neonatal brain parenchyma using 2-dimensional shear wave elastography. J Ultrasound Med. (2018) 37(4):959–67. 10.1002/jum.14366 28850723

[15] Dirrichs T Meiser N Panek A Trepels-Kottek S Orlikowsky T Kuhl CK. Transcranial shear wave elastography of neonatal and infant brains for quantitative evaluation of increased intracranial pressure. Invest Radiol. (2019) 54(11):719–27. 10.1097/RLI.0000000000000602 31464808

[16] Faure F Alison M Francavilla M Boizeau P Guilmin Crepon S Lim C. Transfontanellar shear wave elastography of the neonatal brain for quantitative evaluation of white matter damage. Sci Rep. (2024) 14(1):11827. Published 2024 May 23. 10.1038/s41598-024-60968-w 38782968 PMC11116529

[17] Zhang C Qian L Zhao H. Elucidation of regional mechanical properties of brain tissues based on cell density. Comput Intell Neurosci. (2025) 2025:1–12. 10.1007/s42235-021-0047-6

[18] Weickenmeier J De Rooij R Budday S Steinmann P Ovaert TC Kuhl E. Brain stiffness increases with myelin content. Acta Biomater. (2016) 42:265–72. 10.1016/j.actbio.2016.07.040 27475531

[19] Kuroiwa T Ueki M Ichiki H Kobayashi M Suemasu H Taniguchi I. Time course of tissue elasticity and fluidity in vasogenic brain edema. Acta Neurochir Suppl. (1997) 70:87–90. 10.1007/978-3-7091-6837-0_27 9416287

[20] Kreft B Bergs J Shahryari M Danyel LA Hetzer S Braun J. Cerebral ultrasound time-harmonic elastography reveals softening of the human brain due to dehydration. Front Physiol. (2021) 11:616984. Published 2021 Jan 11. 10.3389/fphys.2020.616984 33505319 PMC7830390

[21] Dent CL Spaeth JP Jones BV Schwartz SM Glauser TA Hallinan B. Brain magnetic resonance imaging abnormalities after the Norwood procedure: a study using regional cerebral perfusion. J Thorac Cardiovasc Surg. (2006) 131:190–7. 10.1016/j.jtcvs.2005.10.003 16399311

[22] Ortinau CM Shimony JS. The congenital heart disease brain: prenatal considerations for perioperative neurocritical care. Pediatr Neurol. (2020) 108:23–30. 10.1016/j.pediatrneurol.2020.01.002 32107137 PMC7306416

[23] McGetrick ME Riviello JJ. Neurological injury in pediatric heart disease: a review of developmental and acquired risk factors and management considerations. Semin Pediatr Neurol. (2024) 49:101115. 10.1016/j.spen.2024.101115 38677794

[24] Mahle WT Tavani F Zimmerman RA Nicolson SC Galli KK Gaynor JW. An MRI study of neurological injury before and after congenital heart surgery. Circulation. (2002) 106(12 Suppl 1):I109–14. 10.1161/01.cir.0000032908.33237.b1 12354718

[25] Wang J Zhang Z Xu X Lu X Wu T Tong M. Real-time shear wave elastography evaluation of the correlation between brain tissue stiffness and body mass index in premature neonates. Transl Pediatr. (2021) 10(12):3230–6. 10.21037/tp-21-513 35070837 PMC8753477

[26] Xu ZS Lee RJ Chu SS Yao A Paun MK Murphy SP. Evidence of changes in brain tissue stiffness after ischemic stroke derived from ultrasound-based elastography. J Ultrasound Med. (2013) 32(3):485–94. 10.7863/jum.2013.32.3.485 23443189

[27] Stroh JN Foreman B Bennett TD Briggs JK Park S Albers DJ. Intracranial pressure-flow relationships in traumatic brain injury patients expose gaps in the tenets of models and pressure-oriented management. Front Physiol. (2024) 15:1381127. Published 2024 Aug 12. 10.3389/fphys.2024.1381127 39189028 PMC11345185

[28] Zhang G Chen C Ren X Zhao Y Ouyang M Billot L. Effects of intensive blood pressure lowering on brain swelling in thrombolyzed acute ischemic stroke: the ENCHANTED results. Stroke. (2025) 56(6):1388–95. 10.1161/STROKEAHA.124.049938 40177745

[29] Cesaro E Saliba T Simoni P. The use of shear-wave elastography for the assessment of muscle spasticity in patients with cerebral palsy, a systematic review. J Clin Ultrasound. (2024) 52(7):923–38. 10.1002/jcu.23706 38708803

[30] Amlerova Z Chmelova M Anderova M Vargova L. Reactive gliosis in traumatic brain injury: a comprehensive review. Front Cell Neurosci. (2024) 18:1335849. Published 2024 Feb 28. 10.3389/fncel.2024.1335849 38481632 PMC10933082

[31] El-Ali AM Subramanian S Krofchik LM Kephart MC Squires JH. Feasibility and reproducibility of shear wave elastography in pediatric cranial ultrasound. Pediatr Radiol. (2020) 50(7):990–6. 10.1007/s00247-019-04592-1 31863191

[32] McIlvain G Schneider JM Matyi MA McGarry MDJ Qi Z Spielberg JM. Mapping brain mechanical property maturation from childhood to adulthood. Neuroimage. (2022) 263:119590. 10.1016/j.neuroimage.2022.119590 36030061 PMC9950297

[33] Hochstetler A Lehtinen MK. Choroid Plexus as a mediator of CNS inflammation in multiple sclerosis. Mult Scler. (2024) 30(5_suppl):19–23. 10.1177/1352458524129297 39503321 PMC11634642

[34] Oishi K Mori S Donohue PK Ernst T Anderson L Buchthal S. multi-contrast human neonatal brain atlas: application to normal neonate development analysis”. Neuroimage. (2011) 56:8. 10.1016/j.neuroimage.2011.01.051 21276861 PMC3066278

[35] Herba CM Roza SJ Govaert P Van Rossum J Hofman A Jaddoe V. Infant brain development and vulnerability to later internalizing difficulties: the generation R study”. J Am Acad Child Adolesc Psychiatry. (2010) 49:1053. 10.1016/j.jaac.2010.07.003 20855050

[36] Ge GR Song W Giannetto MJ Rolland JP Nedergaard M Parker KJ. Mouse brain elastography changes with sleep/wake cycles, aging, and Alzheimer’s disease. Neuroimage. (2024) 295:120662. 10.1016/j.neuroimage.2024.120662 38823503 PMC11409907

